# Skin thickness affects the result of tuberculin skin test in systemic sclerosis

**DOI:** 10.1186/s41927-022-00278-8

**Published:** 2022-08-13

**Authors:** Apichart So-ngern, Ajanee Mahakkanukrauh, Siraphop Suwannaroj, Ratanavadee Nanagara, Chingching Foocharoen

**Affiliations:** Department of Medicine, Faculty of Medicine, Khon Kaen University, Khon Kaen, 40002 Thailand

**Keywords:** Systemic sclerosis, Scleroderma, Diagnostic study, Tuberculin skin test, Mycobacterium tuberculosis, Latent tuberculosis

## Abstract

**Background:**

Skin thickness is a prominent clinical feature of systemic sclerosis (SSc), but there is no consensus on the cut-off for a positive tuberculin skin test (TST) size and the limitation of the TST for a diagnosis of tuberculosis in SSc. We aimed to identify the cut-off size of an indurated TST and the sensitivity and specificity of the test for the diagnosis of tuberculosis in SSc patients.

**Methods:**

A cross-sectional study of 168 adult Thai SSc patients was conducted. The TST was done using 0.1 ml of purified protein derivatives via intradermal injection. The test was interpreted 72 h after testing.

**Results:**

The median age was 57.2 years. The majority (71.8%) had the diffuse cutaneous SSc subset. All the patients had a BCG vaccination at birth, and 17 (10.1%) had a tuberculosis infection. An indurated skin reaction size of 20 mm had the highest specificity for tuberculosis (99.3%: 95%CI 96.4–100) (ROC 0.53). The skin thickness—assessed using the modified Rodnan skin score (mRSS)—had a significant negative correlation with the reaction size (Rho -0.23; p = 0.003).

**Conclusion:**

The TST is not sufficiently sensitive for detecting TB infection in SSc patients, albeit a skin induration of ≥ 15 mm indicates a high specificity for tuberculosis infection. A high mRSS resulted in a smaller skin reaction size when using the TST, which has limited utility as a diagnostic for tuberculosis among SSc patients with severe skin thickness.

The manuscript was presented as a poster presentation at the Annual European Congress of Rheumatology EULAR 2019 Madrid 12–15 June 2019. (Ann Rheum Dis. 2019;78(suppl 2): abstract FRI0347) http://dx.doi.org/10.1136/annrheumdis-2019-eular.1456

**Supplementary Information:**

The online version contains supplementary material available at 10.1186/s41927-022-00278-8.

## Key points

- TST is a noninvasive tool for TB diagnosis, but it is not sensitive for TB diagnosis in SSc.
- Extensive skin thickness affects skin reaction size by TST.

## Introduction

Systemic sclerosis (SSc) is a rare disease for which skin thickness is the hallmark. Skin thickness is the classical presentation in the indurative phase of the disease, and the extent of skin thickness is classified into two major subsets: limited cutaneous systemic sclerosis (lcSSc) and diffuse cutaneous systemic sclerosis (dcSSc) [1]. The skin thickness of lcSSc is limited to the face, hands, feet, and forearms, whereas the skin thickness of dcSSc extends to the trunk and both extremities proximal to the elbows. The modified Rodnan skin score (mRSS) is widely used for evaluating the severity and extent of skin thickness. The current study revealed that ultrasonography might have benefits over mRSS in earlier skin thickness detection [2]. Rapid skin thickness progression was reported in dcSSc patients [3], and this rapid progression is associated with a poor survival outcome and development of a scleroderma renal crisis in the first two years of the disease [3].

Systemic sclerosis affects the thickness of the skin of the trunk and extremities and includes internal organ involvement (interstitial lung disease—ILD). Early pulmonary pathology is active alveolitis characterized by a ground glass appearance, particularly in the lower lobe, and occasional mediastinal lymphadenopathy as visualized using high-resolution computed tomography of the chest (HRCT). Other HRCT findings include a bilateral ground-glass appearance with and without inside fibrosis in the upper and lower lobe [4]. The common presentations include non-productive cough and dyspnea on exertion. Some patients develop a low-grade fever, weight loss, and hypoxemia. These symptoms are not distinguishable from chronic infections like tuberculosis, for which Thailand is an endemic area.

Mediastinal lymphadenopathy is not a common presentation of active alveolitis, and *Mycobacterium tuberculosis* (TB) infection should be excluded. Widely available, initial steps in an investigation include *(a)* a sputum study for acid-fast staining, *(b)* polymerase chain reaction (PCR) for tuberculosis, and *(c)* mycobacterial culture. However, a non-productive cough is a limitation for getting an adequate specimen. Bronchoscopy—an invasive procedure—can be helpful, and bronchoalveolar lavage (BAL) is used to increase the diagnostic yield when a TB infection is suspected [5]. Endobronchial ultrasound (EBUS) transbronchial needle aspiration (TBNA) is an advanced technique for mediastinal node sampling. EBUS is not widely available, and it requires an experienced specialist. Finally, hypoxemia—particularly for extensive lung involvement—makes it difficult to perform invasive procedures, so diagnosis of TB in SSc remains challenging.

The overall incidence of TB in Thailand was 150 cases per 100,000. [World Health Organization, Global Tuberculosis Report. 2020]. TB diagnosis requires microbiological confirmation of infection by sputum smear for acid-fast staining, mycobacterium culture, or caseous granuloma from tissue pathology.

The tuberculin skin test (TST) is a screening tool for detecting occult or remote tuberculosis infection. The TST is simple and inexpensive. It is performed via intradermal injection of PPD 0.1 ml; the result is interpreted 48–72 h after injection.

Although skin thickness is the classical manifestation in SSc, there are no reports on the limitations of performing TST in SSc nor defining the cut-off for a positive TST test size for a diagnosis of TB for such patients. We thus sought to *(a)* investigate the probability of using the TST as a screening tool for TB and *(b)* define the cut-off for a positive TST size in SSc. The results would be applied for *(a)* SSc patients who need immunosuppressants but who should be tested for TB prior to starting therapy, and *(b)* patients needing TB treatment or prophylaxis.

Our objectives were to *(a)* determine the indurated size reaction of the TST and *(b)* define the cut-off TST indurated size, the sensitivity, and specificity of the test for diagnosing TB in SSc patients.

## Method

A cross-sectional study was conducted between November 1, 2016, and November 30, 2017. The TST was performed in SSc patients ≥ 18 years of age at the Scleroderma Clinic, Khon Kaen University, Thailand. All of the patients had a diagnosis of systemic sclerosis (SSc)—based on the American College of Rheumatology criteria and/or fulfilled the classification criteria of systemic sclerosis by the ACR/EULAR 2013 [6]. SSc was classified as the limited or diffuse-type as per LeRoy et al. [7]. To meet the trifecta gold standard, the history of a previous or recent TB infection was reviewed for all patients. We excluded the patients who underwent a TST but for whom the result could not be interpreted within 72 h. We used a simple random sampling technique to choose the patients in the study. Each patient was assigned a sequential number, and the patients with an even number underwent the TST.

## Intervention

**Fig. 1:**
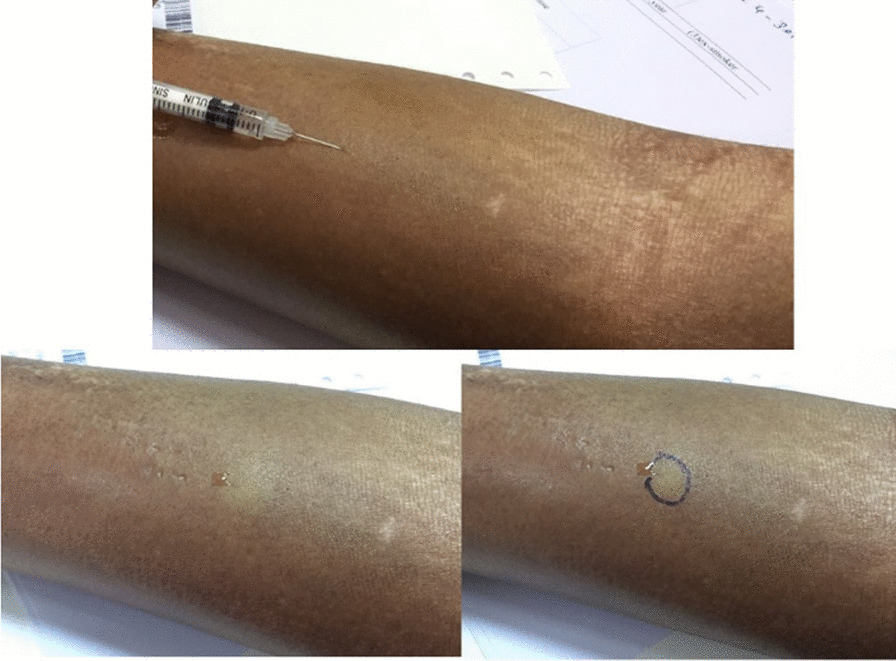
Tuberculin skin test in a systemic sclerosis patient

The TST was performed using purified protein derivatives (PPD) 0.1 ml injected intradermally 5 cm from the cubital fossa (Fig. 1). The PPD 0.1 ml comprised 10 IU of 0.5% phenol and 0.005% Tween 80 in isotonic phosphate buffer saline (PPDTRC of Chiron, Italy). The TST was interpreted by measuring the indurated size 72 h after injection. The test was done and reported by either a trained nurse or doctor.

## Operational definitions

A diagnosis of SSc was based on the American College of Rheumatology (ACR) criteria [8] and/or the 2013 ACR/European League Against Rheumatism (EULAR) Classification Criteria for Scleroderma [6]. SSc was classified as the limited or diffuse-type as per LeRoy et al. [7].

TB was defined by any tissue or fluid culture positive for *Mycobacterium tuberculosis*, positive for acid-fast staining, or a histopathological finding of caseous granuloma. Previous TB infection was defined by a history of TB infection before study enrollment. TB contact was defined as living in the same household as someone with TB.

The onset date was the date when the patients had any SSc symptoms. ILD was defined when either the chest radiograph or HRCT detected interstitial fibrosis. Pulmonary arterial hypertension was defined when the mean pulmonary artery pressure was ≥ 25 mmHg by right heart catheterization [9]. Renal impairment was defined as serum creatinine (Cr) > 1.4 mg/dL. Steroid administration was divided into three groups according to the prednisolone equivalency: *(a)* high dose was > 30 mg/day; *(b)* moderate dose > 7.5 but ≤ 30 mg/day; and, *(c)* low dose ≤ 7.5 mg/day [10]. Immunosuppressants included cyclophosphamide, methotrexate, mycophenolate mofetil, or azathioprine.

## Statistical analysis

The continuous data were presented as means and standard deviations, while the categorical data were presented as proportions or percentages. We set the cut-off skin reaction size of TST according to the classification of the TST reaction of the CDC (Centers for Disease Control and Prevention) into three groups: ≥ 5 mm, ≥ 10 mm, and ≥ 15 mm. We categorized a TST reaction size ≥ 20 mm to another group. The TST reaction size was classified into four groups. The respective sensitivity, specificity, and receiver operating characteristic (ROC) of each cut-off size was calculated. Spearman’s correlation was used to explore the correlation between indurated reaction size and the clinical or laboratory parameters. The data were analyzed using STATA version 11.2 (StataCorp., College Station, TX, USA).

The authors designed the study, and the Human Research Ethics Committee of Khon Kaen University reviewed and approved the study as per the Helsinki Declaration and the Good Clinical Practice Guidelines (HE611617). All eligible patients signed informed consent before enrollment. The sponsor had no role in the study.

## Results

A total of 168 SSc patients were enrolled. The female to male ratio was 1.8:1. The respective median age and the median duration of disease was 57.2 years (IQR 52.5–63.9) and 6.4 years (2.9–10.1). Most patients (71.8%) had the dcSSc subset. Seventeen cases (10.1%) were defined as TB infection, with 16 having had a previous history of infection and 1 having had a recent infection. Before enrollment, the median duration of TB infection was 6.5 years (IQR 3.5–16.9). Of the 151 patients who had a non-tuberculosis infection, 16 cases (10.6%) had TB contact with a relative. All of the patients had a history of BCG vaccination at birth. The results of TST classified by a history of TB infection are presented in Table 1. The TST of those who had had a recent TB infection was no skin reaction. The clinical characteristics of the patients with a previous history of TB infection and no history of infection are presented in Table 2.

**Table 1:** Results of the tuberculin skin test

Indurated size 72 h after the TST															
Tuberculosis infection (N = 17)								Non-tuberculosis infection (N = 151)							
Previous tuberculosis (N = 16)				Recent tuberculosis (N = 1)				Tuberculosis contact (N = 16)				No tuberculosis contact (N = 135)			
< 5 mm	5–9 mm	10–14 mm	≥ 15 mm	< 5 mm	5–9 mm	10–14 mm	≥ 15 mm	< 5 mm	5–9 mm	10–14 mm	≥ 15 mm	< 5 mm	5–9 mm	10–14 mm	≥ 15 mm
N = 14	N = 1	N = 0	N = 1	N = 1	N = 0	N = 0	N = 0	N = 14	N = 2	N = 0	N = 0	N = 117	N = 10	N = 4	N = 4

TST tuberculin skin test, mm millimeter

**Table 2:** The clinical characteristics of the patients with previous history of TB infection and no history of infection

Clinical characteristics	No history of TB infection N = 151	History of TB infection N = 17	p-value
Female (%)	98 (64.9)	9 (52.9)	0.33
dcSSc subset (%)	109 (72.2)	12 (70.6)	0.86
Age at onset (years); mean ± SD	50.9 ± 10.5	50.6 ± 11.2	0.86
Age on study date (years); mean ± SD	57.9 ± 10.0	58.5 ± 11.2	0.89
Duration of disease (years); mean ± SD	6.9 ± 5.4	7.9 ± 6.4	0.53
Serology			
Anti-topoisomerase I antibody positive (%)	77 of 92 (83.7)	10 of 12 (83.3)	0.94
Anti-centromere antibody positive (%)	23 of 57 (40.4)	2 of 8 (25.0)	0.47
BMI ≤ 18.5 kg/m^2^ (%)	35 (23.2)	6 (35.3)	0.28
Clinical characteristics of SSc			
Raynaud’s phenomenon (%)	69 (45.7)	9 (52.9)	0.57
Digital ulcer (%)	28 (18.5)	4 (23.5)	0.62
Telangiectasia (%)	43 (28.5)	9 (52.9)	0.04*
Calcinosis cutis (%)	6 (4.0)	1 (5.9)	0.71
Salt and pepper skin (%)	60 (39.7)	5 (29.4)	0.41
Edematous skin (%)	8 (5.3)	2 (11.8)	0.29
Tendon friction rub (%)	20 (13.3)	2 (11.8)	0.86
Hand deformity (%)	65 (43.1)	7 (41.2)	0.88
Synovitis (%)	13 (8.6)	0	0.21
Muscle weakness (%)	7 (4.6)	0	0.36
Esophageal involvement (%)	77 (60.0)	11 (64.7)	0.28
Stomach involvement (%)	19 (12.6)	4 (23.5)	0.21
Intestinal involvement (%)	13 (8.6)	1 (5.9)	0.70
Interstitial lung disease (%)	77 (51.0)	8 (47.1)	0.76
Pulmonary arterial hypertension (%)	15 (9.9)	1 (5.9)	0.59
Renal crisis (%)	2 (1.3)	0	0.63
mRSS > 20 (%)	20 (13.3)	2 (11.8)	0.86
Treatment			
Prednisolone (%)	84 (55.7)	8 (47.1)	0.77
Low dose (%)	36 (23.8)	3 (17.7)	
Moderate dose (%)	48 (31.8)	5 (29.4)	
High dose (%)	0	0	
Immunosuppressant (%)	45 (29.8)	5 (29.4)	0.96
Methotrexate (%)	12 (7.9)	2 (11.8)	
Cyclophosphamide (%)	24 (15.9)	3 (17.7)	
Mycophenolate mofetil (%)	8 (5.3)	0	
Azathioprine (%)	1 (0.7)	0	

TB tuberculosis; dcSSc diffuse cutaneous systemic sclerosis; SD standard deviation; mRSS modified Rodnan skin score; IQR interquartile range

**Fig. 2:**
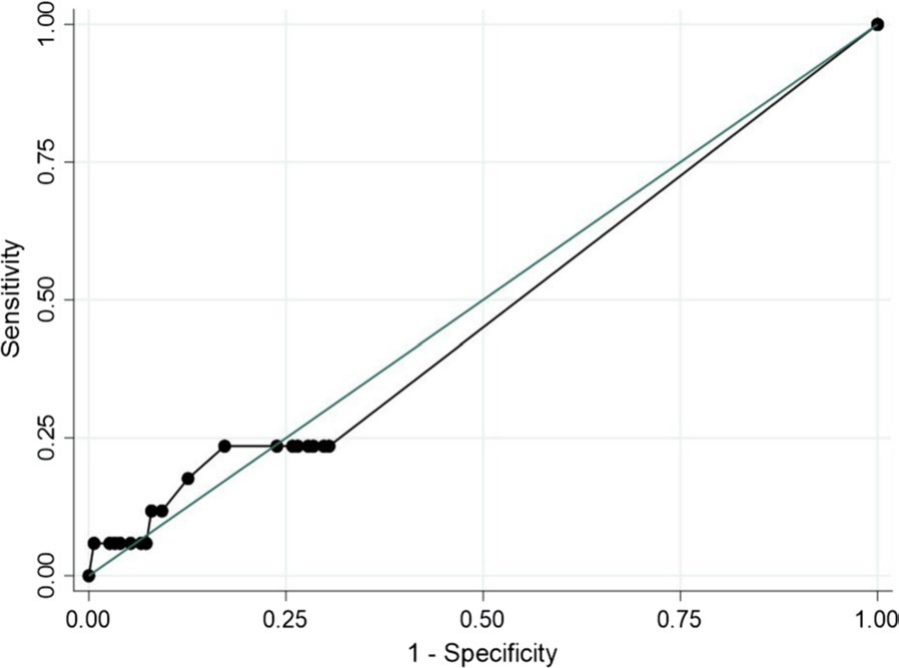
ROC of the TST for diagnosing TB infection

The sensitivity and specificity of the TST for diagnosing TB infection—classified by the size of an indurated skin reaction—were inferred (Table 3). Accordingly, an indurated skin reaction size of 20 mm had a sensitivity of 5.9% (95%CI 0.1–28.7) and a specificity of 99.3% (95%CI 96.4–100) with a ROC of 0.53 (Fig. 2).

**Table 3:** Sensitivity and specificity of the tuberculin skin test for diagnosing a tuberculosis infection

Cut-off indurated reaction size by TST	≥ 5 mm	≥ 10 mm	≥ 15 mm	≥ 20 mm
Sensitivity	17.6%	5.9%	5.9%	5.9%
Specificity	87.4%	94.7%	97.4%	99.3%
ROC area	0.53	0.50	0.52	0.53
Positive likelihood ratio	1.40	1.11	2.22	8.88
Negative likelihood ratio	0.94	0.99	0.97	0.95
Positive predictive value	13.6%	11.1%	20.0%	50.0%
Negative predictive value	90.4%	89.9%	90.2%	90.4%

TST tuberculin skin test, mm millimeters, ROC Receiver operating characteristic

After evaluating the effect of clinical characteristics on the results of the TST, the mRSS had a significant negative correlation with the indurated skin reaction size (Rho − 0.23; p = 0.003). While other clinical parameters—BMI, SSc subset, mRSS of the right forearm where TST was performed, serum albumin level, steroid, or immunosuppressant use—were not correlated with the TST result (p = 0.06, 0.14, 0.19, 0.09, 0.23 and 0.89, respectively).

Eighty-eight of the patients were on a low to moderate steroid dose; 13 of whom had TB infections. After evaluating the effect of steroid use on the TST reaction size, steroid at a low to moderate dose was not significantly related to the indurated skin reaction size (≥ 5 mm, ≥ 10 mm, ≥ 15 mm, and ≥ 20 mm with a p-value of 0.15, 0.13, 0.78, and 0.29, respectively). Likewise, there was no statistically significant association between the indurated skin reaction size and immunosuppressant use (an indurated skin reaction size of ≥ 5 mm, ≥ 10 mm, ≥ 15 mm, and ≥ 20 mm had a p-value of 0.84, 0.24, 0.78, and 0.34, respectively). Overall, 49 patients received immunosuppressants, of whom 9 had TB infections.

## Discussion

The TST is not a routine investigation for TB infection; however, it is helpful for the identification of remote or occult TB infection, particularly in persons at risk of tuberculosis infection (i.e., those who need immunosuppressant therapy or have a history of close contact with persons with TB). TB infection is an infection that has been detected in SSc patients [11, 12]. The diagnosis of pulmonary tuberculosis infection is difficult in SSc patients with previous chest symptoms related to their underlying SSc disease. Our study reported the possible role of the TST for TB infection detection in SSc, which skin thickness might limit TST evaluation.

The respective sensitivity and specificity of the results depends on the community area, history of BCG vaccination, and host. The specificity is high in persons who have not had the BCG vaccination (97%; 95%CI 95–99) [13]. Jamil et al. reported the respective sensitivity and specificity—with a cut-off of ≥ 10 mm—for active pulmonary TB in persons having had a BCG vaccination was 86% and 74% versus 77% and 74% in persons with extrapulmonary TB [14]. Yaacob et al. reported a respective sensitivity and specificity of the TST for active pulmonary TB of 86% and 90% [15]. A positive TST is not necessarily due to active TB but can simply indicate exposure to Mycobacterium infection as the test cannot differentiate between a latent and an active TB infection.

According to our analysis, the TST is not sufficiently sensitive for detecting TB infection in SSc patients. Eighty percent of the SSc patients had a TST skin reaction size of < 5 mm despite having a previous history of infection or recent TB. The sensitivity of the TST in our study was lower than in previous studies;^5,6^ this may be due to the specific SSc population and the difference in the prevalence of TB infection in the general population of other countries. Most of the previous studies represented general populations suspected of having TB infection, where the prevalence of TB infection was 18–24%. Our study population comprised SSc patients with a prevalence of TB of 10%, which is lower than in previous reports. The severity of skin thickness negatively correlated with indurated skin reaction in our SSc patients, so the skin reaction for the TST for TB infection in SSc patients was not apparent as presumed. The severity of the skin thickness can be a factor that affects the sensitivity of the TST. According to the results, we do not recommend using the TST as a screening tool for diagnosing TB infection among patients with SSc. The sensitivity of other test methods (i.e., IGRA) should be investigated as a screening tool for diagnosing TB infection.

False positives and negatives are limitations of the TST. A false positive can occur in persons who have had the BCG vaccination and/or are infected with a non-tuberculosis mycobacterium [16]. Meanwhile, false negatives have been reported in children, the elderly, persons with severe hypoalbuminemia, disseminated TB, and persons with HIV [16, 17]. A meta-analysis by Wang et al. included 26 published articles on the TST and revealed that a previous BCG vaccination was associated with a positive result (Relative risk 2.12, 95%CI 1.50–3.00) compared to persons without a BCG vaccination [18]. The effect of BCG on the TST is reported to be no greater than 10–15 years after vaccination, so false positives are uncommon in communities with a low prevalence of TB infection [19]. Skin indurations of > 15 mm are more likely to indicate a TB infection (remote, recent, or active TB infection) than a BCG vaccination [18, 20]. In general, an indurated skin reaction size of > 10 mm is considered TST-positive in children and adults; however, a reaction size of < 10 mm does not exclude TB [21].

Our findings reveal that a TST skin reaction size of ≥ 15 mm is suggestive of a TB infection (specificity 97.4%). Notably, the sensitivity was low (around 6%), and around 3% (4 from 151 cases) of patients with no history of TB contact and/or no TB infection also had a skin reaction size of ≥ 15 mm. The false-positive TST among those with no history of TB contact and/or with no TB infection might be explained by the effect of the BCG vaccination, but we did not have a TST comparison with a control of ‘no BCG vaccination’. Since all of our SSc patients received a BCG vaccination at birth—in compliance with the national immunization program—our interpretations are limited to SSc patients who had had a BCG vaccination.

Extensive skin thickness—evaluated using the mRSS—affected the TST indurated skin reaction. The majority of our SSc patients were the dcSSc subtype, and our study demonstrated that a high mRSS was negatively correlated with the indurated reaction size to the TST (Rho -0.23, p = 0.003). The mRSS at the right forearm, where TST was performed, did not significantly influence the outcome of the TST skin reaction size. The skin reaction size according to the TST would not be due to local skin thickness but to the overall severity of the disease.

The skin reaction to the TST is a cell-mediated delayed hypersensitivity (Type IV) reaction mediated by skin macrophages, monocytes, and T cells. T cells—sensitized by a previous TB infection—move to the skin test site and release lymphokines. T cells— particularly CD4 + T cells—play a role in the reaction. The study of TST in adults infected with HIV revealed that the patients who were TST-positive had higher CD4 + T cell counts than those who were negative [22]. CD4 + T cells also take part in the pathogenesis of SSc. Activated CD4 + T cells have been reported in the skin, lung, and stomach in SSc patients, and the cells express pathogenic cytokines in both serum and tissue of SSc patients [23, 24].

Despite CD4 + T cells being detected in the skin of SSc patients, the skin reaction to the TST was not as pronounced as expected, particularly in patients who had extensive skin thickness. Therefore, the finding presumes that CD4 + T cells in the skin of SSc patients will respond to some antigen in the skin and cannot move or release the lymphokines related to a TB infection. Nevertheless, the role of CD4 + T cells in the mechanism of cell-mediated delayed hypersensitivity in SSc remains unclear. Therefore, further research is needed to test the hypothesis and study the sensitivity of TST among patients with the lcSSc subset and those who have less skin thickness.

Current steroid use did not influence our SSc patients’ skin reaction size after the TST. We know that steroids can suppress the TST reaction [25], and the reaction size is negatively associated with a higher dose of steroid treatment [26]. Another study found that low-dose steroid treatment did not influence the skin reaction size of the TST among patients with rheumatoid arthritis [27]. Most of our patients received a low to moderate dose of steroid because we wanted to avoid a steroid-induced renal crisis; this might explain why steroid did not affect skin reaction size to the TST in SSc patients. We conclude that low-dose steroid did not influence TST reaction size in SSc patients as it does in rheumatoid arthritis patients. This result gave us confidence in interpreting the TST in SSc patients receiving low-dose steroid during the evaluation. Owing to the unavailability of data on the cumulative dose and the duration of steroid treatment, we cannot comment on their respective contribution to the effect on the TST in SSc patients.

There is currently no test established for the definite diagnosis of occult or latent TB infection. The TST, however, can demonstrate whether or not there is an immune reaction response to a previous mycobacterium exposure. The cut-off skin reaction size for the TST for diagnosis of latent TB infection has been classified according to the risk of exposure and the risk of developing active TB based on the WHO guidelines [28]. Since—by our analysis—skin thickness affects the skin reaction size of the TST, the skin reaction size among SSc patients might not be interpreted as in the general population or even according to the WHO guidelines. Even though some patients had a history of TB contact, we cannot determine the cut-off skin reaction size, the sensitivity, or specificity for diagnosing latent TB infection among SSc patients.

Interferon-gamma assay (IGRA) is another screening tool for diagnosing both latent [29] and active TB infection [30]. Although a study showed the concordance between TST and IGRA results [31], the accuracy of TB infection tests trended to improve when using IGRA (i.e., there was a low rate of false positives) [16, 32]. IGRA is more reliable than the TST in cases of BCG vaccination because a BCG vaccination does not confound the IGRA [31]. In addition, the cross-reactivity with non-tuberculosis Mycobacterium infection is low with the IGRA test [16]. Notwithstanding, there is limited data on IGRA testing in various settings and populations, so the results must be interpreted in concurrence with clinical, radiography, and risk assessment [32]. Moreover, the IGRA is much more expensive than the TST and is available at only a few centers, while the TST is widely available.

The current study had some limitations: *(a)* the low prevalence of TB infection in patients with SSc, which would affect the sensitivity of the test; *(b)* the absence of patients without BCG vaccination, so the sensitivity and specificity of the TST in such patients could not be determined; *(c)* the lack of a comparison of the sensitivity and specificity of the TST among SSc patients with/without the BCG vaccination; *(d)* we cannot provide the TST reaction after boosting in patients who received immunosuppressants due to a shortage of tuberculin and the crossectional study design; and, *(e)* because of financial limitations, none of our patients received biological DMARDs, so we cannot conclude whether biological DMARDs affect TST reaction. The strengths of our study were *(a)* the use of the TST, which is a widely available, feasible, and practical tool for diagnosing TB infection in resource-limited areas, making the results of value to daily practice; and, *(b)* the inclusion of parameters of interest, particularly local skin thickness where the TST would be injected. The severity of skin thickness and current steroid/immunosuppressant therapy can influence the TST skin size reaction in SSc. The findings of our study are valuable for evaluating TB infection among SSc patients and for planning future studies.

## Conclusion

The TST is not sensitive for the detection of tuberculosis infection in SSc. A skin induration of ≥ 15 mm indicated a high specificity for tuberculosis infection. As thickness determined by the mRSS, high skin thickness resulted in a smaller skin reaction size when using the TST. The TST is thus less useful for diagnosing tuberculosis among SSc patients, especially those with severe skin thickness.

## Supplementary Information

**Additional file 1: Table S1**. Data of systemic sclerosis patients with tuberculin skin test.

## Data Availability

All data generated or analyzed during this study are included in the supplementary information file (Additional file 1).

## References

[1] Silver RM (1991). Clinical aspects of systemic sclerosis (scleroderma). Ann Rheum Dis.

[2] Santiago T, Santiago M, Ruaro B, Salvador MJ, Cutolo M, da Silva JAP (2019). Ultrasonography for the assessment of skin in systemic sclerosis: a systematic review. Arthritis Care Res (Hoboken).

[3] Domsic RT, Rodriguez-Reyna T, Lucas M, Fertig N, Medsger TA (2011). Skin thickness progression rate: a predictor of mortality and early internal organ involvement in diffuse scleroderma. Ann Rheum Dis.

[4] Orlandi M, Landini N, Sambataro G, Nardi C, Tofani L, Bruni C (2022). The role of chest CT in deciphering interstitial lung involvement: systemic sclerosis versus COVID-19. Rheumatol (Oxf).

[5] Ahmad M, Ibrahim WH, Sarafandi SA, Shahzada KS, Ahmed S, Haq IU (2019). Diagnostic value of bronchoalveolar lavage in the subset of patients with negative sputum/smear and mycobacterial culture and a suspicion of pulmonary tuberculosis. Int J Infect Dis.

[6] van den Hoogen F, Khanna D, Fransen J, Johnson SR, Baron M, Tyndall A (2013). 2013 classification criteria for systemic sclerosis: an american college of Rheumatology/European league against Rheumatism collaborative initiative. Arthritis Rheum.

[7] LeRoy EC, Black C, Fleischmajer R, Jablonska S, Krieg T, Medsger TA (1988). Scleroderma (systemic sclerosis): classification, subsets and pathogenesis. J Rheumatol.

[8] Masi AT (1980). Preliminary criteria for the classification of systemic sclerosis (scleroderma) Subcommittee for scleroderma criteria of the american rheumatism association diagnostic and therapeutic criteria committee. Arthritis Rheum.

[9] Mukerjee D, St George D, Knight C, Davar J, Wells AU, Du Bois RM (2004). Echocardiography and pulmonary function as screening tests for pulmonary arterial hypertension in systemic sclerosis. Rheumatology (Oxford).

[10] Buttgereit F, da Silva JAP, Boers M, Burmester G-R, Cutolo M, Jacobs J (2002). Standardised nomenclature for glucocorticoid dosages and glucocorticoid treatment regimens: current questions and tentative answers in rheumatology. Ann Rheum Dis.

[11] Foocharoen C, Nanagara R, Foocharoen T, Mootsikapun P, Suwannaroj S, Mahakkanukrauh A (2010). Clinical features of tuberculous septic arthritis in Khon Kaen, Thailand: a 10-year retrospective study. Southeast Asian J Trop Med Public Health.

[12] Foocharoen C, Siriphannon Y, Mahakkanukrauh A, Suwannaroj S, Nanagara R (2012). Incidence rate and causes of infection in Thai systemic sclerosis patients. Int J Rheum Dis.

[13] Pai M, Zwerling A, Menzies D (2008). Systematic review: T-cell-based assays for the diagnosis of latent tuberculosis infection: an update. Ann Intern Med.

[14] Jamil B, Qamruddin S, Sarwari A (2008). An assessment of mantoux test in the diagnosis of tuberculosis in a BCG-vaccinated, tuberculosis endemic area. J Infect Dis Parkistan.

[15] Yaacob I, Ahmad Z (1990). Clinical significance of Mantoux test in Malaysian patients. Med J Malaysia.

[16] Gong W, Wu X (2021). Differential diagnosis of latent tuberculosis infection and active tuberculosis: A key to a successful tuberculosis control strategy. Front Microbiol.

[17] Liam C, Lim K, Wong C (1998). Changes in serum proteins, erythrocyte sedimentation rate and Mantoux tuberculin skin test reactivity in active tuberculosis. JUMMEC.

[18] Wang L, Turner MO, Elwood RK, Schulzer M, FitzGerald JM (2002). A meta-analysis of the effect of bacille calmette guérin vaccination on tuberculin skin test measurements. Thorax.

[19] Araujo Z, de Waard JH, de Larrea CF, Borges R, Convit J (2008). The effect of bacille calmette-guérin vaccine on tuberculin reactivity in indigenous children from communities with high prevalence of tuberculosis. Vaccine.

[20] Farhat M, Greenaway C, Pai M, Menzies D (2006). False-positive tuberculin skin tests: what is the absolute effect of BCG and non-tuberculous mycobacteria?. Int J Tuberc Lung Dis.

[21] Rose DN, Schechter CB, Adler JJ (1995). Interpretation of the tuberculin skin test. J Gen Intern Med.

[22] Rodríguez JI, Arias M, París SC, Arbeláez MP, Betancur J, García LF (1997). Tuberculin skin test and CD4+/CD8+ T cell counts in adults infected with the human immunodeficiency virus in Medellín. Colombia Mem Inst Oswaldo Cruz.

[23] Balbir-Gurman A, Braun-Moscovici Y (2012). Scleroderma - new aspects in pathogenesis and treatment. Best Pract Res Clin Rheumatol.

[24] Manetti M, Neumann E, Müller A, Schmeiser T, Saar P, Milia AF (2008). Endothelial/lymphocyte activation leads to prominent CD4+ T cell infiltration in the gastric mucosa of patients with systemic sclerosis. Arthritis Rheum.

[25] Agarwal S, Das SK, Agarwal GG, Srivastava R (2014). Steroids decrease prevalence of positive tuberculin skin test in rheumatoid arthritis: Implications on Anti-TNF therapies. Interdiscip Perspect Infect Dis.

[26] Schatz M, Patterson R, Kloner R, Falk J (1976). The prevalence of tuberculosis and positive tuberculin skin tests in a steroid-treated asthmatic population. Ann Intern Med.

[27] Reitblat O, Lerman TT, Cohen O, Reitblat T (2018). The effect of prednisone on tuberculin skin test reaction in patients with rheumatoid arthritis. Int J Rheumatol.

[28] Guidelines on the Management of Latent Tuberculosis Infection. Geneva: World Health Organization; 2015. 25973515

[29] Moon H-W, Hur M (2013). Interferon-gamma release assays for the diagnosis of latent tuberculosis infection: an updated review. Ann Clin Lab Sci.

[30] Metcalfe JZ, Everett CK, Steingart KR, Cattamanchi A, Huang L, Hopewell PC (2011). Interferon-γ release assays for active pulmonary tuberculosis diagnosis in adults in low- and middle-income countries: systematic review and meta-analysis. J Infect Dis.

[31] Abdel-Samea S, Ismail Y, Fayed S, Mohammad A (2013). Comparative study between using QuantiFERON and tuberculin skin test in diagnosis of Mycobacterium tuberculosis infection. EJCT.

[32] Hamada Y, Cirillo DM, Matteelli A, Penn-Nicholson A, Rangaka MX, Ruhwald M (2021). Tests for tuberculosis infection: landscape analysis. Eur Respir J.

